# Variation of fruit phenotypic traits of *Cinnamomum camphora* from 11 provenances in China

**DOI:** 10.1371/journal.pone.0319877

**Published:** 2026-05-20

**Authors:** Shengying Wan, Jiao Zhao, Jie Ma, Jie Zhang, Qingqing Liu, Changlong Xiao, Jiexi Hou, Zhinong Jin

**Affiliations:** 1 Jiangxi University of Water Resources and Electric Power, Nanchang, Jiangxi, China; 2 Jiangxi Provincial Engineering Research Center of Seed-Breeding and Utilization of Camphor Trees, Nanchang, Jiangxi, China; Southwest Jiaotong University, CHINA

## Abstract

This study aimed to characterize the diversity and variation of fruit phenotypic traits in natural provenances of *Cinnamomum camphora* to facilitate the selection of superior germplasm. According to a provenance-based sampling design, 10 fruit phenotypes were investigated across 170 individuals from 11 provenances from Jiangxi province, China. The results showed that the average phenotypic differentiation coefficients among and within provenances were 73.82% and 26.18%, respectively, indicating that variation among provenances was the main source of phenotypic diversity in *C. camphora* in Jiangxi province, China. The mean Shannon-Wiener index (H) for the fruit phenotypic traits of *C. camphora* was 2.62, with the high diversity observed in fruit horizontal diameter/fruit vertical diameter (FHD/FVD, 2.848), fruit thousand-grain weight/fruit volume (FTW/FV, 2.835) and the fruit side diameter/fruit horizontal diameter (FSD/FHD, 2.817). The coefficients of variation of fruit phenotypic traits among different provenances ranged from 2.64% to 25.45%, with the largest value for peel thickness (PT). Furthermore, significant or highly significant correlations existed among fruit phenotypic traits. There was a significant correlation among fruit phenotypic traits of *C. camphora* and latitude, longitude, climate factors in different provenances of Jiangxi province. Based on the results of principal component analysis and cluster analysis, the cumulative contribution rate reached 78.78%, and the fruit side diameter (FSD), fruit side diameter/fruit vertical diameter (FSD/FVD), fruit side diameter/fruit horizontal diameter (FSD/FHD) could comprehensively reflect the information and ranking of the 10 traits. The fruit phenotypic traits from subgroup B2 were bigger. These selected individuals can be used as basic materials for variety improvement and conservation. The high phenotypic diversity indicates a rich genetic base, suggesting that future studies should employ molecular markers to assess genetic diversity and identify associated genes.

## Introduction

*Cinnamomum camphora* (Lauraceae) is a subtropical evergreen tree species of significant economic and ecological value, integrating timber production, landscaping, medicinal applications, and biochemical functions into its utility [1–4]. Jiangxi renowned as “the hometown of camphor wood” in China, represents a modern distribution center for this species and conserves abundant natural resources resulting from millennia of cultivation history [5]. Despite this rich germplasm foundation, strategic conservation and genetic improvement programs in Jiangxi remain markedly underdeveloped.

The comprehensive collection and preservation of germplasm resources demand substantial investment of human, financial, and material resources [6–7], underscoring the critical need for efficient screening strategies to optimize conservation and utilization efficiency [8]. Plant phenotypic traits, which arise from the interplay of genetic and environmental factors, serve as primary indicators for assessing diversity and preliminary selection [9–11]. Considerable studies on diverse tree species, including *Idesia polycarpa* Maxim. (Salicaceae) [12], *Elaeagnus mollis* Diels. (Elaeagnaceae) [13], *Canarium album* L. (Burseraceae) [14] and *Melia azedarach* (Meliaceae) [15] have demonstrated substantial provenance-based variation in fruit traits, providing a critical basis for elite germplasm selection. These phenotypic differences were frequently correlated with geographic and climatic factors, such as longitude, latitude and temperature [16–17].

Despite the ecological and economic importance of *C. camphora* in Jiangxi, a significant knowledge gap exists regarding the patterns of fruit phenotypic diversity and its relationship with geographical gradients in this region [18]. Furthermore, it remains unknown which specific fruit traits are most influential for discriminating among provenances and selecting superior germplasm, and is geographically limited to regions such as Sichuan and Zhejiang provinces in China. The patterns of fruit phenotypic variation in Jiangxi which is a region critical to the species distribution remain completely unexplored.

While assessing genetic diversity using molecular markers provides the most direct measure of heritable variation [19], these approaches can be cost-prohibitive and technically demanding for initial screening of large germplasm collections. Therefore, the evaluation of phenotypic diversity remains a fundamental and cost effective first step for identifying promising germplasm and capturing potential genetic variation, upon which subsequent molecular analyses can be focused.

Therefore, to address this knowledge gap, this study investigated the fruit phenotypic diversity of 170 *C. camphora* individuals sampled from 11 natural provenances across Jiangxi province. We aimed to quantify the diversity and differentiation of fruit traits, elucidate their relationships with geographical and climatic factors, and identify superior germplasm resources. Our findings would provide a scientific basis for breeding selection and the conservation of *C. camphora* germplasm resources.

## Materials and methods

### Study area and plant materials

**Fig 1:**
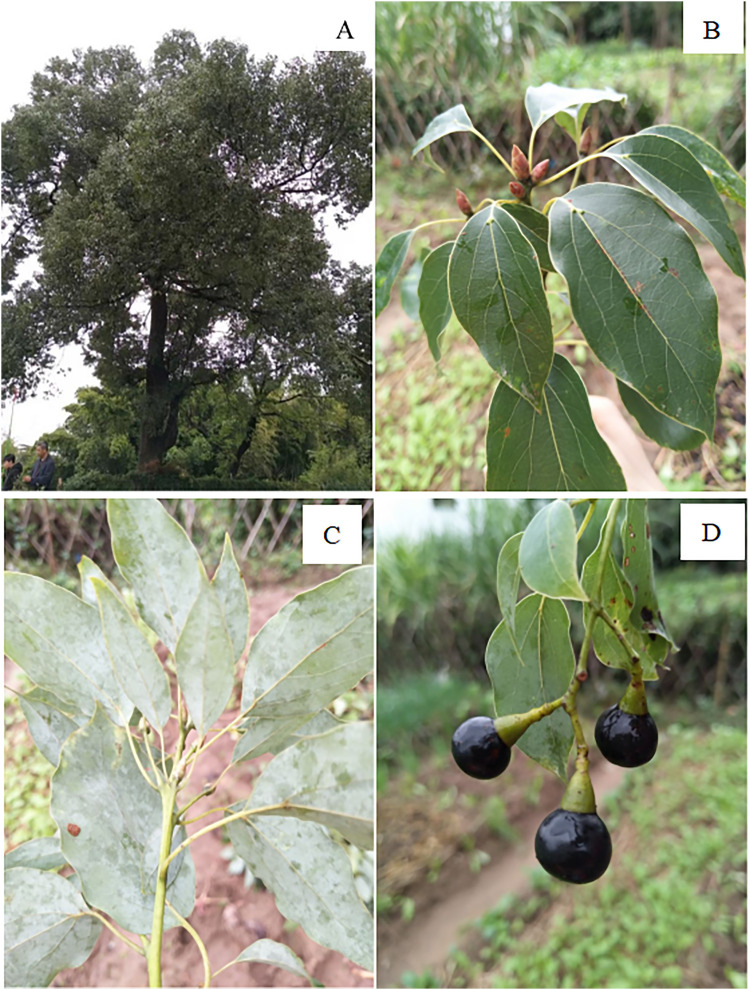
Different parts of *C. camphora.* (A) Overall plant morphology; (B) The front (adaxial) side of a leaf; (C) The back (abaxial) side of a leaf; (D) Fruit.

A total of 170 healthy and vigorous plants showing no visible signs of disease or pest infestation and with intact canopy architecture were selected. The fruit collection was conducted in December 2018 across 45 counties within 11 cities of Jiangxi province, China, with official permission from Jiangxi provincial Department of Forestry. The geographic location information and meteorological data from the monitoring information of meteorological stations of 11 provenances were shown in Table 1. Jiangxi province has a subtropical monsoon climate. The annual average sunshine duration is 1447.98–1805.13 h, and the mean altitude is 17.16–388 m. The annual mean temperature is 17.65–18.98 ℃. The annual mean precipitation is 1461.50–1898.14 mm, and the soil is dominated by red soil. Several normal-growing *C. camphora* fruits with a diameter at breast height greater than 60 cm were collected using a systematic sampling method at 2 km intervals to ensure spatial independence of samples and effectively capture intra provenance variation [20]. The organs of the plant were presented in Fig 1.

**Table 1:** Geographic location information and sampling of *C. camphora* in different provenances.

Provenance	Gathering area	Longitude(E)	Latitude(N)	Annual average sunshine duration (h)	Mean temperature in January (℃)	Mean temperature in July (℃)	Mean altitude (m)	Annual mean temperature (℃)	Annual mean precipitation (mm)	Sampling number	Number
**NC**	Nanchang	115°46’	28°43’	1705.17	5.47	29.87	65	18.12	1644.99	7	1-7
	Jinxian	116°20’	28°25’								
**YT**	Yingtan	117°11’	28°16’	1645.89	6.48	30.1	170	18.72	1898.14	3	8-10
**SR**	Shangrao	117°50’	28°21’	1699.33	5.84	29.45	327	18.10	1825.20	30	11-40
	Hengfeng	117°31’	28°29’								
	Yushan	118°17’	28°47’								
	Yiyang	117°26’	28°23’								
	Yugan	116°25’	28°52’								
	Dexing	116°37’	28°49’								
	Poyang	116°38’	29°19’								
	Wannian	115°23’	29°12’								
**JJ**	Duchang	116°20’	29°12’	1805.13	4.43	28.85	17.16	17.16	1461.50	16	41-56
	Pengze	116°36’	29°56’								
	Hukou	116°21’	29°40’								
	De’an	115°41’	29°19’								
**YC**	Yichun	114°21′	27°45′	1593.49	5.46	28.88	242	17.65	1707.79	27	57-83
	Fengxin	115°11’	28°43’								
	Gaoan	115°22’	28°26’								
	Shanggao	114°56’	28°17’								
	Wanzai	114°27’	28°03’								
	Tonggu	114°31’	28°39’								
	Zhangshu	115°19’	28°04’								
**JDZ**	Leping	117°9’	28°56’	1801.06	5.7	29.54	32	18.11	1826.68	7	84-90
	Fuliang	117°30′	29°29′								
**PX**	Shangli	113°47′	27°47′	1447.98	5.51	28.96	274	17.68	1624.70	11	91-101
	Pingxiang	113°52′	27°34′								
	Luxi	114°03′	27°38′								
**XY**	Yushui	114°50′	27°55′	1628.64	6.01	29.73	103	18.37	1603.09	4	102-105
**JA**	Xiajiang	115°11′	27°34′	1564.38	6.06	29.22	268	18.14	1564.38	19	106-124
	Yongfeng	115°21′	27°22′								
	Jishui	115°15′	27°13′								
	Wanan	114°46′	26°28′								
	Yongxin	114°18′	27°1′								
**FZ**	Jinxi	116°39’	28°47′	1600.60	6.13	29.27	271	18.23	1793.21	15	125-139
	Nanfeng	116°38′	27°19′								
	Lichuan	116°57′	27°23′								
	Nancheng	116°45′	27°51′								
**GZ**	Xingguo	115°14′	26°32′	1627.88	8.24	28.19	388	18.98	1576.02	31	140-170
	Ningdu	115°51′	26°26′								
	Shangyou	114°30′	25°47′								
	Nankang	114°38′	25°49′								
	Dayu	114°21′	25°22′								
	Dingnan	115°20′	24°43′								
	Anyuan	115°22′	25°60′								
	Xunwu	115°44′	24°58′								

Notes: NC, Nanchang; YT, Yingtan; SR, Shangrao; FZ, Fuzhou; JJ, Jiujiang; YC, Yichun; JDZ, Jingdezhen; JA, Ji’an; PX, Pingxiang; XY, Xinyu; GZ, Ganzhou.

### Data collection

In this study, approximately 2 kg of mature fruits were collected from the four cardinal directions (east, south, west and north) of each tree. From this bulk sample, one hundred fruits per tree (n = 100) were randomly selected for the measurement of the fruit thousand-grain weight (FTW, g) and fruit volume (FV, cm³). The fruit thousand-grain weight (FTW, g) was measured using an electronic balance with an accuracy of 0.1 g, while the fruit volume (FV, cm³) was determined by the water displacement method. First, we added an appropriate amount of water to a graduated cylinder and recorded the initial volume (V_1_, mL), then slowly and completely immersed the fruit in the water, ensuring no air bubbles adhere to it. After the liquid stabilizes, the final volume (V_2_, mL) was recorded. The fruit volume was calculated as the difference between the two readings (V = V_2_-V_1_, mL). If the fruit floated, it was submerged fully before taking the reading.

Additionally, thirty fruits per tree (n = 30) were randomly chosen to measure the following morphological traits of fruit vertical diameter (FVD, mm), fruit horizontal diameter (FHD, mm), fruit side diameter (FSD, mm) and peel thickness (PT, mm), using an electronic vernier caliper with an accuracy of 0.01 mm. The fruit vertical diameter (FVD, mm) was defined as the maximum distance from the top to the base of the longitudinal section, the fruit horizontal diameter (FHD, mm) as the maximum distance from the ventral suture line to the opposite side in the transverse section, and the fruit side diameter (FSD, mm) as the maximum distance across both sides of the ventral suture line [21]. Three replicates were performed for each trait.

Finally, the following of fruit side diameter/fruit horizontal diameter (FSD/FHD), fruit side diameter/fruit vertical diameter (FSD/FVD), fruit horizontal diameter/fruit vertical diameter (FHD/FVD) and fruit thousand-grain weight/fruit volume (FTW/FV, g·cm^-3^) were calculated based on the above measurements.

### Data and statistics

Microsoft Excel 2023 was used to examine the variation of fruit phenotypic traits, including mean value, standard error and coefficient of variation. SPSS27.0 software, minitab software and PAST software were used to analyze the diversity of each phenotype, including multiple comparisons, analyzed using one-way analysis of variance (ANOVA) and regression analysis. Means comparison method were conducted both among and within provenances, and one way ANOVA was performed to assess the differences among provenances. When ANOVA revealed significant effects, means were separated by Duncan’s new multiple range test at a significance level of *p* < 0.05. And Origin 2024 software was further used for principal component analysis and cluster analysis of fruit phenotypes in 11 provenances.

The formula CV=$\frac{s}{\overset{―}{x}}$ ×100% was used to calculated the coefficient of variation, where CV was the coefficient of variation, $\overset{―}{\text{x}}$was the average value of phenotypic trait, *s* was the trait standard deviation [22].

Shannon-Wiener index was a quantitative representation of the variation of trait diversity. The calculation formula was H = $-\sum \begin{array}{c} n\\ i=1 \end{array}pilnpi$. In the formula, H was the diversity index, and *pi* was the effective percentage of the distribution frequency within the material at level *i* of a trait [23].

The formula V_ST_= [δ_2t/S_/(δ_2t/S_ + δ_2S_)] × 100% was used to calculated the variation of phenotypic differentiation coefficient among provenances. V_ST_ was the phenotypic differentiation coefficient, representing the percentage of among provenances of total genetic variation. δ_2t/S_ was variance component among provenances, and δ_2S_ was variance component within provenances. And the sum of variation of phenotypic differentiation coefficient among and within provenances was one [24].

## Results

### Variation of fruit phenotypic traits of *C. camphora* in different provenances

The variation of fruit phenotypic traits was shown in Table 2. The differences among provenances of FTW, FV, FHD, PT, FSD/FHD and FSD/FVD reached a extremely significant level (*P* < 0.01). There were significant differences of FTW, FSD/FHD, FSD/FVD within provenances (*P* < 0.05). The mean variance component among provenances (23.87%) was greater than that within provenances (10.44%). The phenotypic differentiation coefficient among provenances ranged from 37.04% to 94.45% across all traits, with all values exceeding 30%. The highest value was observed for FVD (94.45%), while the coefficients for FV and FSD were all above 80%. In contrast, the phenotypic differentiation coefficient within provenance ranged from 5.55% to 62.96%. It was indicated that variation among provenances was the main source in *C. camphora* in Jiangxi province, China.

**Table 2:** Comparison of phenotypic traits and phenotypic differentiation coefficients of *C. camphora* fruits from different provenances.

Fruit phenotypic character	Among provenances					Within provenances					Random error	
	Mean square	F value	Variance component(%)	Phenotypic differentiation coefficient (%)	Df	Mean square	F value	variance component (%)	Phenotypic differentiation coefficient (%)	Df	Mean square	Heft (%)
**FTW**	30683.95	4.11**	16.81	65.69	10	8094.2	1.09*	8.78	34.31	159	7462.92	74.41
**FV**	29588.82	4.21**	17.98	83.17	10	7550.89	1.08ns	3.64	16.83	159	7027.19	78.37
**FSD**	0.66	1.72ns	17.72	84.06	10	0.32	0.84ns	3.36	15.94	159	0.38	78.92
**FHD**	0.70	2.30**	16.14	72.57	10	0.28	0.92ns	6.10	27.43	159	0.31	77.76
**FVD**	0.65	1.84ns	27.51	94.45	10	0.25	0.72ns	1.61	5.55	159	0.35	70.80
**PT**	0.26	3.76**	34.84	80.82	10	0.09	1.23ns	8.27	19.18	159	0.07	56.89
**FSD/FHD**	0.00	3.28**	23.24	77.86	10	0.00	1.34*	6.61	22.14	159	0.00	70.15
**FSD/FVD**	0.00	3.25**	17.94	66.34	10	0.00	1.26*	9.10	33.65	159	0.00	72.96
**FHD/FVD**	0.00	2.62ns	17.85	76.15	10	0.00	0.10ns	5.59	23.85	159	0.00	76.56
**FTW/FV**	0.01	9.88ns	48.69	37.04	10	0.00	1ns	51.31	62.96	159	0.00	62.93
**Average**	6027.51	3.70	23.87	73.82	10	1564.60	0.96	10.44	26.18	159	1449.12	71.98

Notes: * and ** indicate significant difference at *P* < 0.05 and *P* < 0.01, ns indicates not significant, respectively.

FTW, Fruit thousand-grain weight; FV, Fruit volume; FSD, Fruit side diameter; FHD, Fruit horizontal diameter; FVD, Fruit vertical diameter; PT, Peel thickness.

Significant differences were observed in fruit phenotypes of *C. camphora* among 11 provenances in Jiangxi, China (Table 3). The traits exhibited considerable variation, with ranges of 454.9–608.74 g for FTW and 438.19–586.43 cm³ for FV. The JJ provenance recorded the lowest values for both these traits.

**Table 3:** Comparison on fruit phenotypic characters of *C. camphora* from different provenances and analysis on Shannon-Wiener index.

Provenance	FTW (g)	FV (cm^3^)	FSD(mm)	FHD(mm)	FVD (mm)	PT (mm)	FSD/FHD	FSD/FVD	FHD/FVD	FTW/FV (g·cm^-3^)
**NC**	488.65bc	468.73bcd	9.07bc	8.20bc	8.76ab	0.98bc	1.11a	1.02ab	0.94c	1.05a
**YT**	478.67bc	451.85 cd	8.91c	8.09c	8.45b	1.02abc	1.10a	1.05ab	0.96abc	1.06a
**SR**	514.26bc	490.85bcd	9.33abc	8.63abc	9.15a	1.27ab	1.08ab	1.02b	0.94bc	1.05a
**FZ**	552.80ab	534.81abc	9.26abc	8.49abc	8.78ab	0.99bc	1.09ab	1.06ab	0.97abc	1.03a
**JJ**	454.94c	438.19d	9.20abc	8.61abc	8.99ab	0.89c	1.07b	1.02b	0.96abc	1.04a
**YC**	570.11ab	546.71ab	9.66ab	8.81a	8.91ab	1.13abc	1.10ab	1.09a	0.99ab	1.03a
**JDZ**	474.57bc	460.32bcd	9.54abc	8.75ab	9.10a	1.28ab	1.09ab	1.05ab	0.96abc	1.03a
**JA**	608.74a	586.43a	9.77a	9.08a	9.36a	1.28ab	1.08ab	1.05ab	0.97abc	1.04a
**PX**	548.27abc	526.36abcd	9.62ab	8.87a	9.27a	1.10abc	1.09ab	1.04ab	0.96abc	1.04a
**XY**	539.75abc	516.66abcd	9.28abc	8.86a	8.93ab	1.28a	1.05c	1.04ab	0.99a	1.04a
**GZ**	565.05ab	544.09abc	9.55abc	8.81a	9.25a	1.13abc	1.08ab	1.03ab	0.95abc	1.04a
**H**	2.395	2.395	2.446	2.450	2.448	2.795	2.817	2.813	2.848	2.835

Notes: Different lower cases in the same column indicate the significant difference at 0.05 level.

H stands for Shannon-Wiener index.

Similarly, the ranges for FSD, FHD, FVD, and PT were 8.91–9.77 mm, 8.09–9.08 mm, 8.45–9.36 mm and 0.89–1.28 mm, respectively. The YT provenance showed the lowest values for FSD, FHD, and FVD. In contrast, the JA provenance consistently exhibited the largest values for FTW, FV, FSD, FHD, FVD and PT among all provenances.

The ranges of FSD/FHD, FSD/FVD, FHD/FVD and FTW/FV were 1.05 to 1.11, 1.02 to 1.09, 0.94 to 0.99 and 1.03 to 1.06 g·cm^-3^, respectively. The largest of FSD/FHD was found in the NC and YT provenances, while the smallest was in XY. The YC provenance had the largest FSD/FVD, and the JJ provenance had the smallest. The XY provenance showed the largest FHD/FVD, and the NC provenance had the smallest. No significant differences were detected among provenances for the FTW/FV.

The Shannon-Wiener index for ten traits ranged from 2.40 to 2.85. The highest diversity was observed in FHD/FVD, while the lowest was in FTW and FV. These results collectively indicated that *C. camphora* fruits from different provenances possessed rich genetic diversity.

Variation coefficient of phenotypic traits in different provenances was shown in Table 4. Among 10 fruit phenotypic traits in different provenances, the largest coefficient of variation was PT (25.45%) followed by FV as the second highest (17.83%), and the smallest coefficient of variation was FSD/FHD (2.64%). According to the average coefficient variation of phenotypic traits in 11 provenances, 2 provenances were more than 10%, among which PX provenance was the largest with the value for 11.14%, followed by NC provenance (10.68%), and YT provenance was the smallest (5.15%). The mean value of the coefficient of variation among provenances (9.65%) was greater than the variation within provenances (8.52%) for fruit phenotypic traits of *C. camphora*.

**Table 4:** Analysis of coefficient of variation of fruit phenotypic traits of *C. camphora* among provenances in Jiangxi province.

Provenances	FTW	FV	FSD	FHD	FVD	PT	FSD /FHD	FSD /FVD	FHD /FVD	FTW/FV	Average
**NC**	18.26	19.68	10.14	10.01	9.65	28.32	3.13	1.23	2.96	3.39	10.68
**YT**	12.04	11.62	4.23	4.90	4.94	6.59	0.84	2.51	3.11	0.69	5.15
**SR**	13.38	13.46	6.53	5.93	5.65	16.55	2.48	4.08	3.33	2.12	7.35
**FZ**	16.54	15.16	6.10	6.00	8.03	22.19	2.88	6.43	5.89	3.06	9.23
**JJ**	15.14	12.85	3.92	3.58	3.32	41.57	1.71	4.04	3.80	4.30	9.42
**YC**	17.54	18.47	7.27	7.65	7.38	25.20	2.46	4.32	4.08	3.99	9.84
**JDZ**	12.32	12.38	6.03	4.05	7.20	8.61	3.18	7.70	6.75	5.91	7.41
**JA**	11.79	11.82	4.71	4.52	6.00	23.11	1.65	5.24	4.38	1.90	7.51
**PX**	24.15	24.89	7.39	7.67	6.12	25.96	3.12	5.08	5.08	1.97	11.14
**XY**	11.49	13.17	4.95	2.98	2.80	22.10	2.00	3.03	1.66	1.24	6.54
**GZ**	16.66	17.03	6.64	6.67	6.04	24.59	2.70	4.74	4.50	4.65	9.42
**CV*/*%**	17.66	17.83	6.64	6.56	6.57	25.45	2.64	5.08	4.56	3.51	9.65

Note: CV indicates the coefficient of variation.

### Correlation analysis on fruit phenotypic traits of *C. camphora* in Jiangxi province

**Fig 2:**
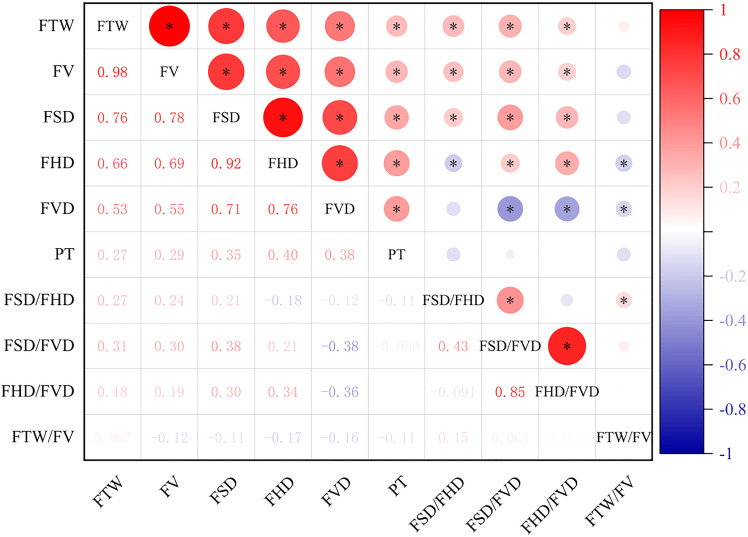
Correlation analysis among 10 fruit phenotypic traits of *C. camphora.* FTW: fruit thousand-grain weight; FV: fruit volume; FSD: fruit side diameter; FHD: fruit horizontal diameter; FVD: fruit vertical diameter; PT: peel thickness; FSD/FHD: ratio of fruit side diameter to horizontal diameter; FSD/FVD: ratio of fruit side diameter to vertical diameter; FHD/FVD: ratio of fruit horizontal diameter to vertical diameter; FTW/FV: ratio of fruit thousand-grain weight to volume. Values are Pearson correlation coefficients ranging from 0 to 1.The color gradient bar on the right (from blue to red) indicates the strength of correlation, with darker red representing stronger positive correlations. *p < 0.05.

There were certain correlations among fruit phenotypic traits of *C. camphora* from different provenances in Jiangxi province, China (Fig 2). There were significant positive correlations between FTW and four traits of FV, FSD, FHD and FVD (*P* < 0.05). FV showed a highly significant correlation with FSD, FHD and FVD. FSD showed significantly positively correlated with FHD and FV. FHD was significantly positively correlated with FV, while negatively correlated with FSD/FHD and FTW/FV. FVD showed significantly positively correlated with PT, while there were significant negative correlations with FSD/FVD, FHD/FVD and FTW/FV. In summary, most of the relevant traits of *C. camphora* fruits were closely related, and when one trait was changed, it might cause changes in other indexes.

### Response of fruit phenotypic traits to latitude, longitude and climatic factors from different provenances

**Fig 3:**
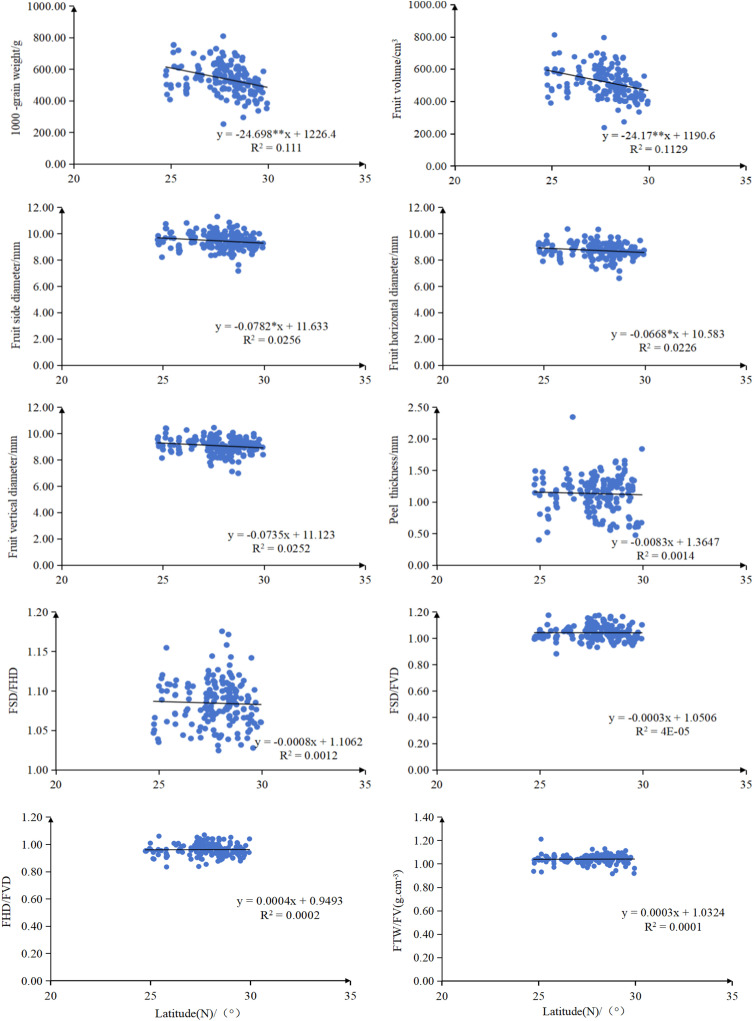
Relationship between fruit phenotypic traits of *C. camphora* from different provenances and latitude. Each panel shows the relationship between one fruit phenotypic trait (y-axis) and latitude (x-axis, °N). Fitted linear regression lines are presented with corresponding regression equations, correlation coefficients (R2), and significance levels (**P < 0.01, *P < 0.05).

**Fig 4:**
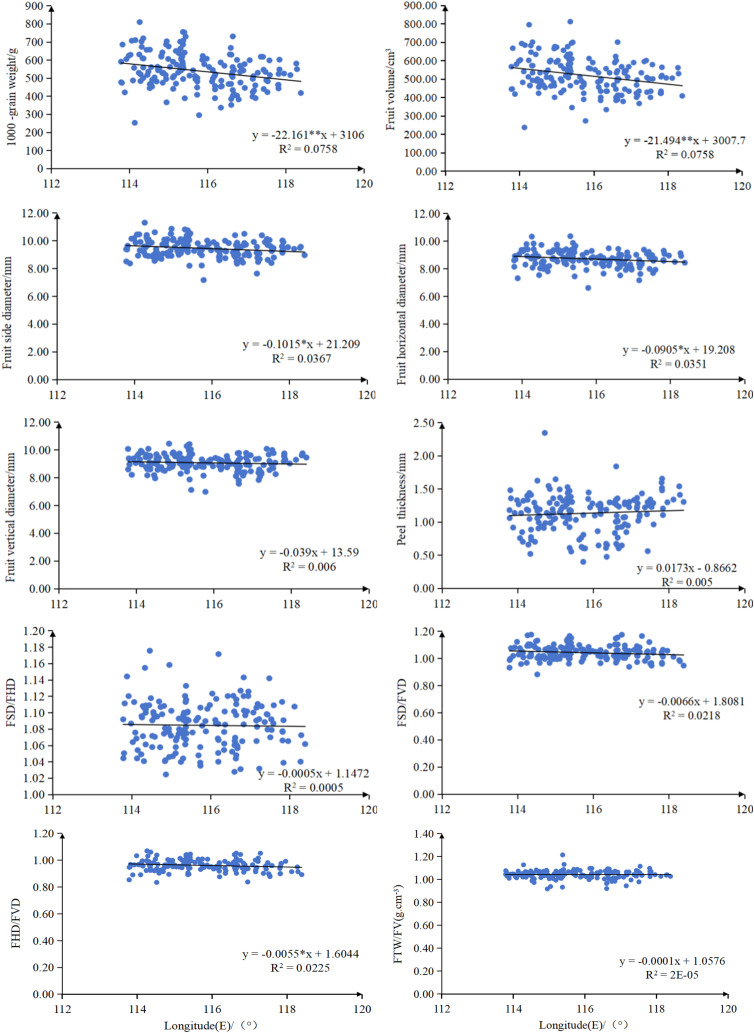
Relationship between fruit phenotypic traits of *C. camphora* from different provenances and longitude. Multiple linear regression lines are shown, with the corresponding regression equations labeled within the figure. The X-axis represents longitude (°); the Y-axis represents fruit phenotypic traits (see labels within the figure or the main text for specific trait names). Each line was fitted based on the original data points; in the equations, x denotes longitude, and y denotes the corresponding fruit phenotypic trait value. Raw data points are not shown in the figure; please refer to the main text or supplementary materials for details.

Regression analysis revealed significant geographic patterns in the phenotypic traits of *C. camphora* different provenances (Fig 3 and Fig 4). Both FTW and FV showed a highly significant negative correlation with latitude (*P* < 0.01). Similarly, FSD and FHD were significantly negatively correlated with latitude (*P* < 0.05). Regarding longitude, FSD, FHD, FSD/FVD and FHD/FVD all exhibited a significant negative correlation (*P* < 0.05), indicating pronounced geographic variation in the phenotypic traits of *C. camphora* in Jiangxi province.

The correlation between fruit phenotypic traits and geo-climatic factors was detailed in Table 5. FTW and FV were positively correlated with the mean temperature in January, mean altitude (*P* < 0.01) and annual mean temperature (*P* < 0.05), but negatively correlated with the annual average sunshine duration (*P* < 0.01) and mean temperature in July (*P* < 0.05). FSD showed a significant negative correlation with annual sunshine duration (*P* < 0.01). FHD was negatively correlated with the mean temperature in July and annual sunshine duration (*P* < 0.05). Furthermore, FVD was positively correlated with mean altitude (*P* < 0.05), while PT was positively correlated with both mean altitude and annual mean precipitation (*P* < 0.05). Among the ratio traits, FSD/FHD was positively correlated with annual precipitation (*P* < 0.05), whereas FSD/FVD was negatively correlated with annual sunshine duration (*P* < 0.05).

**Table 5:** Correlation between fruit phenotypic traits of *C. camphora* and climatic factors.

Fruit phenotypic trait	Annual mean temperature (℃)	Mean temperature in January (℃)	Mean temperature in July (℃)	Annual average sunshine duration (h)	Mean altitude (m)	Annual mean Precipitation (mm)
**FTW**	0.177*	0.22**	−0.16*	−0.37**	0.30**	0.00
**FV**	0.18*	0.22**	−0.17*	−0.37**	0.29**	0.00
**FSD**	0.04	0.08	−0.15	−0.21**	0.13	−0.03
**FHD**	0.02	0.08	−0.16*	−0.17*	0.12	−0.11
**FVD**	0.11	0.14	−0.14	0.11	0.17*	−0.10
**PT**	0.12	0.10	0.09	−0.08	0.19*	0.20*
**FSD/FHD**	0.07	0.02	0.03	−0.08	0.05	0.19*
**FSD/FVD**	−0.09	−0.06	−0.02	−0.17*	−0.04	0.09
**FHD/FVD**	−0.14	−0.08	−0.03	−0.14	−0.07	0.00
**FTW/FV**	−0.01	−0.02	0.05	−0.02	0.04	0.06

Note: * and ** indicate significant difference at *P* < 0.05 and *P* < 0.01, ns indicates not significant, respectively, according to Pearson correlation.

### Principal component analysis of fruit phenotypic traits of different provenances

**Fig 5:**
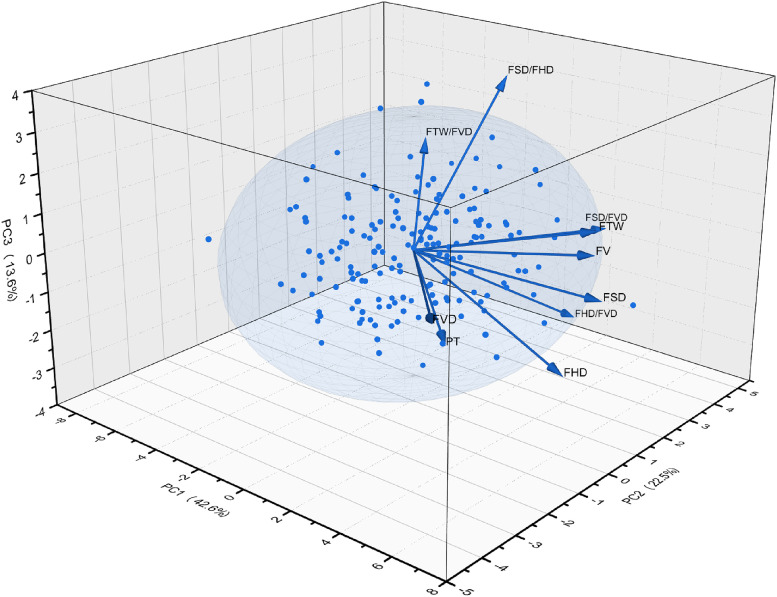
The principal component analysis of *C. camphora* fruit phenotypic traits in different provenances of Jiangxi province. The figure shows a 3D PCA plot. Points represent samples from different provenances; vectors represent individual fruit phenotypic traits. The X-axis (PC1), Y-axis (PC2), and Z-axis (PC3) are the first, second, and third principal components, respectively. For definitions of FTW, FVD, FSD, FHD, and their ratios, please refer to Fig 2.

Principal component analysis was conducted in fruit phenotypic traits from different provenances in Jiangxi province of China, in which three principal components with eigenvalues greater than 1.00 were yielded with a cumulative contribution rate of 78.78%. It was indicated that the three principal components represented most of the information on phenotypic traits of *C. camphora* (Fig 5). Among them, the 1st principal component had the highest contribution rate of 42.6%, with an eigenvalue for 4.26. And eigenvector absolute value of FSD (0.96), FHD (0.91), FV (0.91) and FTW (0.89) were larger, which indicated that the 1st principal component was mainly associated with the traits related to fruit size. The contribution rate of the 2nd principal component was 22.5% with the eigenvalue for 2.255, and the absolute values of eigenvectors in 3 phenotypic traits of FSD/FVD (0.92), FHD/FVD (0.78) and FVD (−0.67) were larger, indicating that the 2^nd^ principal component was related to fruit shape. The contribution rate of the 3^rd^ principal component was 13.6%, and the eigenvalue was 1.361, in which FSD/FHD (0.76) and FHD/FVD (−0.54) had the largest absolute value, indicating that the 3rd principal component was also a trait mainly reflecting fruit shape. In summary, FSD, FSD/FVD and FSD/FHD could comprehensively reflect the information of 10 traits.

According to fruit phenotypic trait load coefficient of *C. camphora* on the three principal components and the contribution rate of the three principal components, the comprehensive score of each *C. camphora* tree was calculated, so as to obtain the top 20 plants of *C. camphora* in the comprehensive score, with the sample number for 79, 67, 112, 166, 72, 137, 98, 165, 142, 120, 66, 159, 80, 97, 87, 11, 59, 135, 109 and 157. And the corresponding scores were 3.08, 2.12, 1.82, 1.81, 1.77, 1.76, 1.60, 1.59, 1.56, 1.53, 1.52, 1.483, 1.47, 1.29, 1.25, 1.24, 1.19, 1.18, 1.15 and 1.12, respectively. Among them, there were 6 plants from YC provenance, 5 plants from GZ provenance, 4 plants from JA provenance, 2 plants from FZ provenance, 2 plants from PX provenance and 1 plant from JDZ provenance (Table 7).

**Table 7:** Principal component scores and comprehensive scores of the top 20 individual *C. camphora* plants with comprehensive scores.

ID	The scores of individual plants on principal components 1st, 2nd and 3rd			Individual plant score	Ranking
	PC1	PC2	PC3		
**79**	6.40	2.17	−0.98	3.08	1
**67**	3.73	0.97	2.32	2.12	2
**112**	3.99	0.50	0.02	1.82	3
**166**	5.33	−1.84	−0.37	1.81	4
**72**	4.03	1.18	−1.54	1.77	5
**137**	2.79	2.54	−0.04	1.76	6
**98**	3.14	0.55	1.01	1.60	7
**165**	3.66	−0.77	1.53	1.59	8
**142**	4.58	−0.87	−1.47	1.56	9
**120**	2.92	0.89	0.59	1.53	10
**66**	3.54	0.27	−0.39	1.52	11
**159**	1.57	3.71	−0.17	1.48	12
**80**	2.50	2.25	−0.77	1.47	13
**97**	2.38	1.64	−0.67	1.29	14
**87**	2.19	2.38	−1.61	1.25	15
**111**	2.10	1.71	−0.33	1.24	16
**59**	2.97	−0.44	0.16	1.19	17
**135**	0.51	3.94	0.54	1.18	18
**109**	1.45	2.42	−0.10	1.15	19
**157**	0.85	1.89	2.42	1.12	20

### Cluster analysis based on fruit phenotypic traits of *C. camphora* from different provenances

**Fig 6:**
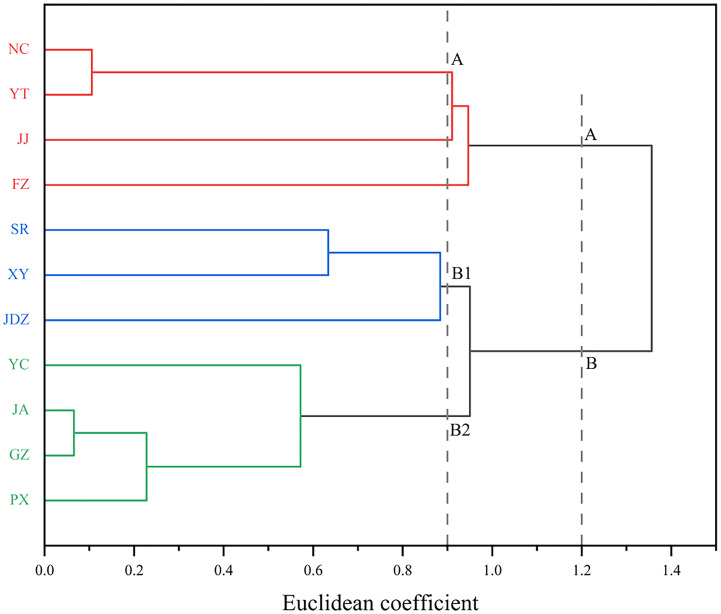
The cluster analysis of fruit traits of *C. camphora* from different provenances. The figure shows a dendrogram based on euclidean distance. The Y-axis represents provenance abbreviations; full names are provided in Table 1. The X-axis represents the euclidean coefficient, indicating the dissimilarity of fruit traits among provenances. Provenances with smaller distances exhibit more similar fruit traits.

Hierarchical cluster analysis was performed based on the Pearson correlation coefficient using the between-groups linkage method to analyze the 10 fruit phenotypic traits of *C. camphora* from 11 provenances in Jiangxi province, China (Fig 6). The mean values of fruit phenotypic traits for each resultant group were calculated (Table 8).

**Table 8:** Comparison of fruit phenotypic traits of different groups of *C. camphora.*

Group	FTW (g)	FV(cm^3^)	FSD(mm)	FHD(mm)	FVD(mm)	PT(mm)	FSD /FHD	FSD /FVD	FHD /FVD	FTW/FV (g·cm^-3^)
**Group**										
**A**	493.72b	473.40b	9.11b	8.35b	8.75b	0.97b	1.09a	1.14a	1.05a	1.04a
**B**	545.79a	525.28a	9.53a	8.83a	9.14a	1.21a	1.08b	1.04b	0.97b	1.04a
**Subgroup**										
**A**	493.73b	473.40b	9.11c	8.35b	8.75b	0.97c	1.09a	1.14a	1.05a	1.05a
**B1**	509.51b	491.13b	9.38b	8.75a	9.06ab	1.28a	1.04a	1.04a	0.96a	1.04a
**B2**	573.00a	550.90a	9.65a	8.89a	9.20a	1.16b	1.05a	1.05a	0.97a	1.04a

The dendrogram showed that *C. camphora* from different provenances could be divided into 2 major groups at a distance coefficient of 1.2. These were further subdivided at a distance coefficient of 0.9, where group A emerged as a distinct cluster, and group B was partitioned into subgroups B1 and B2.

Group A included four provenances (NC, YT, JJ, FZ). Subgroup B1 comprised three provenances (SR, JDZ, XY) and subgroup B2 contained the remaining four (JA, YC, GZ, PX). The fruit phenotypic traits of group B provenances were significantly greater than those of group A, as evidenced by thicker peel, larger fruit size and heavier fruit weight. Furthermore, within group B, FTW, FV and FSD in the B2 provenances were significantly higher than in B1, while PT in B2 was significantly lower than in subgroup B1.

## Discussion

### Diversity and variation of fruit phenotypic traits of *C. camphora*

Our findings revealed exceptionally high phenotypic differentiation among provenances, significantly exceeding that reported for *Xanthoceras sorbifolium* (Sapindaceae) and *Quercus fabri* (Fagaceae) [25–31]. This suggested that local adaptation, rather than gene flow or genetic drift, was the predominant force driving fruit trait diversification in *C. camphora* across Jiangxi’s complex landscape. The substantial geographic isolation and habitat heterogeneity in this region likely imposed strong selective pressures, leading to pronounced genetic divergence among provenances. The moderate to high Shannon-Wiener indices (2.395–2.835) further confirmed that among provenance diversity was preserved despite this strong differentiation, a pattern consistent with that observed in *Zanthoxylum armatum* (Rutaceae) [32–33]. The notably lower diversity in species like *Ziziphus jujuba* Mill. (Rhamnaceae) may reflect their more restricted genetic base or different breeding systems [34–35].

The coefficient of variation provides insights into the stability and evolvability of traits. The highest coefficient of variation observed in PT (25.45%) indicated that this trait was highly responsive to environmental cues or under weaker genetic constraint, making it a potential target for selective breeding for specific fruit characteristics. This aligned with findings by Li et al. [36] and Gao et al. [37]. In contrast, the lower coefficient of variation in dimensional traits like FHD and FVD suggested they were under stronger stabilizing selection or were more canalized developmentally, which was crucial for maintaining basic fruit architecture. The high stability of FSD, FHD and FVD might be a unique feature of our sampled provenances [38–39].

### Geographical variation and clustering results of fruit phenotypic traits in *C. camphora*

The correlation patterns unraveled the complex interplay between genotype and environment. The unexpected positive correlation of FTW, FV and PT with altitude contradicted the common trend of smaller fruits at higher elevations due to resource limitation [40–41]. This anomaly could be explained by our specific altitudinal range (32–388 m). Within this range, increased altitude likely mitigated summer heat stress and amplified diurnal temperature fluctuations, both of which could enhance photosynthetic efficiency and nutrient accumulation, ultimately facilitating larger fruit development [42]. Conversely, the negative correlation with summer temperature and sunshine duration underscored heat stress as a major limiting factor. Excessive sun and heat during Jiangxi’s summer might inhibit metabolic processes crucial for fruit growth [43]. The latitudinal and longitudinal clined further support that gradients in temperature and precipitation, tied to geographic position, were key drivers of phenotypic variation [44].

Principal component analysis efficiently reduced trait dimensionality, identifying FSD, FSD/FVD and FSD/FHD as key integrators of fruit morphology. This suggested that selection on these easily measurable traits could effectively improve multiple fruit characteristics simultaneously, a strategy more efficient than independent culling [45]. Cluster analysis grouped provenances into three distinct clusters, not strictly by geography, implying that microhabitat factors like soil composition and topography were important alongside climate [46]. The B2 cluster, characterized by larger and heavier fruits, represented a prime candidate pool for germplasm conservation and breeding programs aimed at enhancing yield [47]. Building on this, future research should directly link these advantageous phenotypic traits to economically important qualities, such as fruit oil content and nutritional value, as seen in *Xanthoceras sorbifolium* Bunge (Sapindacea) [48] and *Fortunella crassifolia* (Rutaceae) [49]. Furthermore, exploring the potential relationship between leaf essential oil content and fruit phenotypes could enable the non-destructive selection of high oil yielding varieties based on fruit traits, revolutionizing breeding strategies for *C. camphora*.

### Conclusions

In conclusion, our study comprehensively demonstrated the rich diversity in fruit phenotypic traits among natural provenances of *C. camphora* in Jiangxi Province, which primarily originated from differences among provenances and reflected strong local adaptation to heterogeneous environments, as evidenced by significant correlations between traits and geographical-climatic factors. Furthermore, FSD, FSD/FVD, and FSD/FHD were identified as key integrative indicators for efficiently evaluating fruit traits. The superior germplasm resources identified, particularly those from the B2 subgroup characterized by larger fruit size and weight, provide a valuable material base for conservation and breeding. Based on these findings, future research should employ molecular markers to quantify the underlying genetic diversity and provenance structure, thereby establishing a foundation for the screening of germplasm resources.

## Supporting information

S1 File The values behind the means, standard deviations and other measures reported. (XLSX)

S2 File The values used to build graphs. (XLSX)

S3 File The points extracted from images for analysis. (XLSX)

S1 Data Minimal data. (XLSX)
